# Brain and Spinal Cord Tumours of Childhood

**DOI:** 10.1038/sj.bjc.6602349

**Published:** 2005-01-25

**Authors:** G Jallo

**Affiliations:** 1 Johns Hopkins Hospital, Baltimore, USA

Although there are many textbooks on Paediatric Neurosurgery, the editors have assembled a cast of experts in the field of paediatric neurology and neurosurgery. They have edited a modern, concise and comprehensive text on the subject. The book is divided into six parts, with the first two sections dedicated to the historical basis of neurooncology and the epidemiology of childhood brain and spinal cord tumours. These parts contain chapters that explain, in a succinct manner, the epidemiology and tumour biology of central nervous system (CNS) tumours.

Part three represents the diagnosis, both clinical and radiographic, for CNS tumours. These chapters discuss all the imaging modalities for tumours, including MRI, MR spectroscopy and perfusion scans. A unique feature of this text is the tables associated with the chapter. For example, one table focuses on MR sequence recommendations at the time of diagnosis and follow-up imaging. These tables are easy to understand and can serve as reference in the management of children. The authors clearly define tumour response on imaging studies. This is a difficult and controversial topic where many surgeons have their own definition of response (complete, partial, stable or progressive). There is also a chapter about clinical trials, which details the design and rational for oncology trials. This is a most informative chapter for the novice who will better understand the clinical studies for CNS tumours.

Parts four and five concentrate on the treatment techniques for tumours in general and for specific tumour types. The discussion focuses on radiotherapy techniques (Chapter 10) and neuropsychological outcome (Chapter 11). This is a very important topic, which is not discussed in many other textbooks concerning paediatric neurosurgery and neurology. Again is the relatively easy algorithm for paediatric brain tumour outcome (with regard to predictors and time frame).

Part five focuses on the specific tumour types, including the low-grade astrocytomas, high-grade tumours, brainstem tumours, embryonic tumours, ependymomas, germ-cell tumours, infant tumours, rare tumours and a specific chapter on intradural tumours. These chapters are very well written and cohesive in text. The chapter on brainstem tumours is exceptional, it discusses the assessment and care for brainstem tumours in children. There is emphasis on the more common, diffuse pontine glioma. This chapter has several tables, which detail the radiotherapy and chemotherapy protocols for these tumours and outcome. The table is simple and forward, which provides the reader with many different therapies attempted and outcome for this particular type, and has never been outlined prior to this textbook. The textbook has unique chapters, chapter 26 – Cognitive development and educational rehabilitation, and Chapter 28 – Information needs for children and families. These chapters are unique to this textbook. The chapters focus on the impact of CNS tumours on the cognitive and behavioural effects upon children. These authors discuss the planning involved when children return to the school environment. The final chapter is information for children and the caregiver, which also includes web-based resources. Although the listing is not comprehensive, it provides the practitioner ‘internet’ resources in caring for children.

This book represents a comprehensive textbook about paediatric brain and spinal cord tumours. It is an excellent reference book for all practitioners at any level in neurology, neurosurgery and oncology. This textbook has many unique features, which make it ‘a must have’ textbook for all who care for children with CNS tumours.

